# Posterior Epistaxis Presenting as Upper GI Bleeding in A Healthy 21‐Year‐Old Patient: A Case Report

**DOI:** 10.1002/ccr3.70355

**Published:** 2025-03-24

**Authors:** Aref AlRajabi, Bara M. AbuIrayyeh, Amal M. Shawabka, Anwar Yousef Jabari, Sami D. Jabari, Kareem Ibraheem

**Affiliations:** ^1^ Faculty of Medicine Palestine Polytechnic University Hebron Palestine; ^2^ Palestinian Clinical Research Center Bethlehem Palestine

**Keywords:** epistaxis, epistaxis‐induced hematemesis, obscure GI bleeding, posterior nasal bleeding, recurrent gastrointestinal bleeding

## Abstract

A 21‐year‐old Palestinian woman experienced recurrent hematemesis and melena over 7 months, requiring multiple hospital admissions and blood transfusions. Despite extensive investigations, the bleeding source remained undetermined until a posterior nasal bleed was suspected. Flexible rhinoscopy revealed a dilated sphenopalatine artery, and she was successfully treated with endovascular intervention. It is important for the Gastroenterologists to consider nasal endoscopy for patients with unexplained UGIB.

## 1 Introduction

Overt gastrointestinal (GI) bleeding is divided into upper and lower GI bleeding. Hematemesis is the usual symptom of overt UGIB; if the bleeding is abrupt, melena and hematochezia may also occur. Hematochezia is the characteristic symptom of overt LGIB; melena may develop if the bleeding originates in the small intestine or proximal colon [1]. Hemodynamic resuscitation and endoscopic source identification of hemorrhage (and treatment if possible) should be the primary goals of care for a patient with overt GI bleeding [2]. When a patient presents with the symptoms of GI bleeding, the patient goes through investigations to identify the source of bleeding; if the source is not identified after a thorough examination of the GI system, it is called obscure GI bleeding [3]. Less common causes of hematemesis and melena are present and include posterior epistaxis. Although It is a common disorder that typically manifests with clinically evident symptoms, it is important to acknowledge that, albeit rare, it can also manifest with hematemesis and melena [4]. Failure to recognize this possibility can potentially lead to a delay in initiating the appropriate and definitive treatment.

In this case report, we present the perplexing journey of a 21‐year‐old female patient who endured repeated episodes of hematemesis and melena for nearly 7 months. Despite undergoing extensive investigations and multiple hospital admissions for blood transfusions, the source of bleeding remained undetermined. The aim of this report is to shed light on the diagnostic challenges encountered and the eventual discovery of a posterior nasal origin for the bleeding.

## 2 Case Presentation

### 2.1 Case History/Examination

A 21‐year‐old female Palestinian patient presented to the gastroenterology department following a history of multiple episodes of coffee ground emesis and melena, associated with dizziness, exertional dyspnea, and intermittent palpitations. On examination, she was conscious, oriented, and alert, but she looked tired and pale. She was hypotensive (106/60 mmHg) and tachypneic; otherwise, the physical examination, including HEENT, was unremarkable.

## 3 Methods (Differential Diagnosis, Investigations, and Treatment)

The differential diagnosis for the patient's recurrent bleeding episodes included gastrointestinal bleeding, due to her history of peptic ulcer disease, bleeding disorders, or a hematologic malignancy. Initial investigations comprised an upper endoscopy, which revealed normal gastrointestinal mucosa with no identifiable bleeding source. Further workups indicated normochromic normocytic anemia with anisopoikilocytosis, microspherocytes, and polychromatic red blood cells, while a bone marrow biopsy excluded hematolymphoid malignancies. Additional tests, including coagulation profiles, autoimmune panels, and imaging (abdominal ultrasound, CT angiography, chest X‐ray, and abdominal CT), returned normal findings. During an acute hematemesis episode, blood observed in the esophagus suggested a nasal origin. ENT evaluation with flexible rhinoscopy identified a dilated, pulsating sphenopalatine artery. However, it did not localize the bleeding to a single, distinct branch of the SPA. The pulsatile nature of the bleeding suggested the involvement of the entire sphenopalatine artery complex rather than an isolated branch.

Finally, carotid CT angiography was done, revealed a nasal source for the bleeding, and showed no significant stenosis and no obvious aneurysmal dilations.

## 4 Treatment, Outcome and Follow‐Up

**FIGURE 1:**
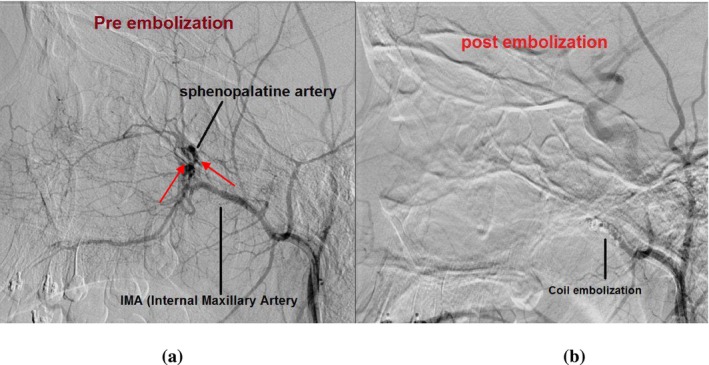
(a) Angiography, lateral view, arterial phase, of the left internal maxillary artery and left sphenopalatine artery(IMA/SPA) showing extravasation (red arrows). (b) post selective embolization of the second and third portions of the left internal maxillary artery with 2 mm coil and gel foam.

Posterior nasal packing was performed for 24 h, and her hemoglobin levels stabilized during this period, but they dropped to 4 g/dL upon removal of the nasal packing. It was decided that she needed a specialized ENT center for further intervention. She subsequently underwent selective embolization of the second and third portions of the left internal maxillary artery with a 2 mm coil and gel foam (Figure 1), leading to improvement and stabilization of her hemoglobin levels, after which she was discharged. Specialists here preferred embolization over SPA ligation as it could be challenging due to the artery's small size, deep location in the nasal cavity, and closeness to important structures, which can lead to incomplete bleeding control or complications. In contrast, embolization is a less invasive and more precise option that effectively stops the bleeding by blocking the internal maxillary artery, preserves nasal anatomy, and avoids risks like tissue damage.

**FIGURE 2:**
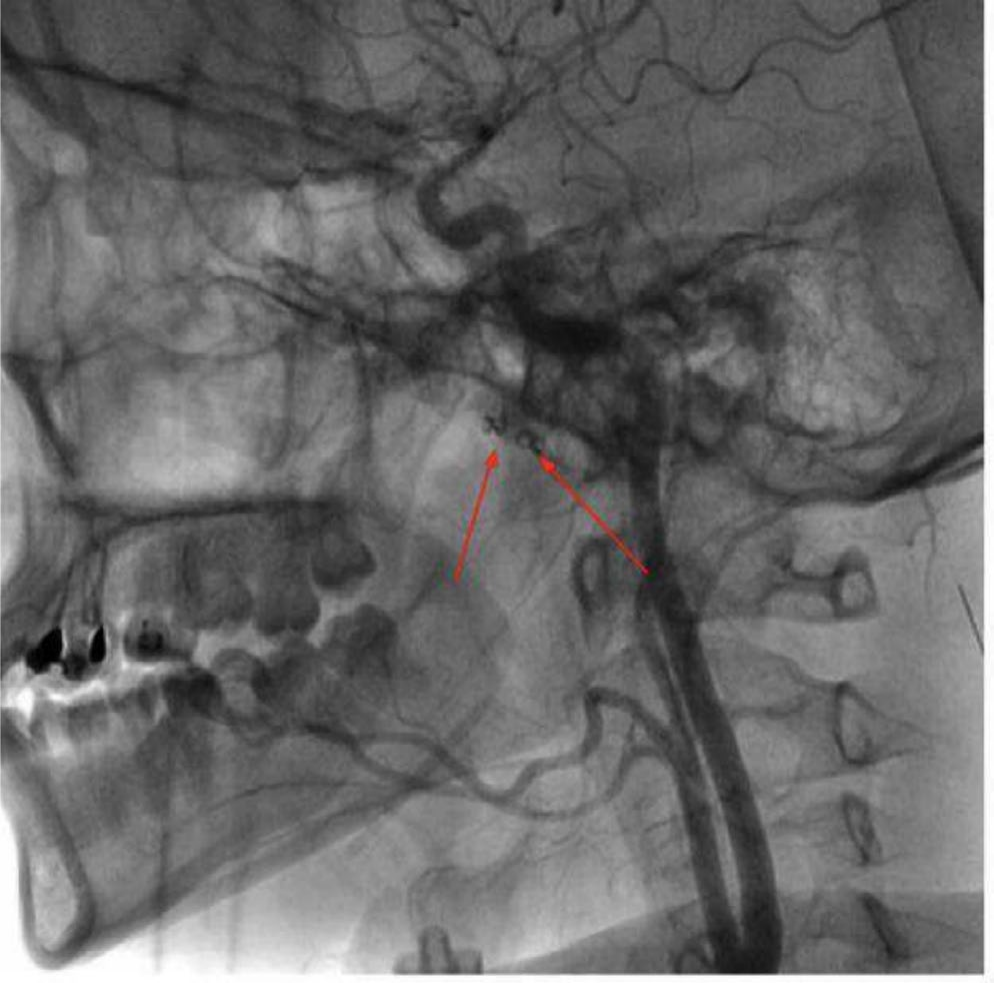
Digital subtraction angiography illustrating the coils that have been placed in the second and third portions of the left internal maxillary artery (red arrows).

**FIGURE 3:**
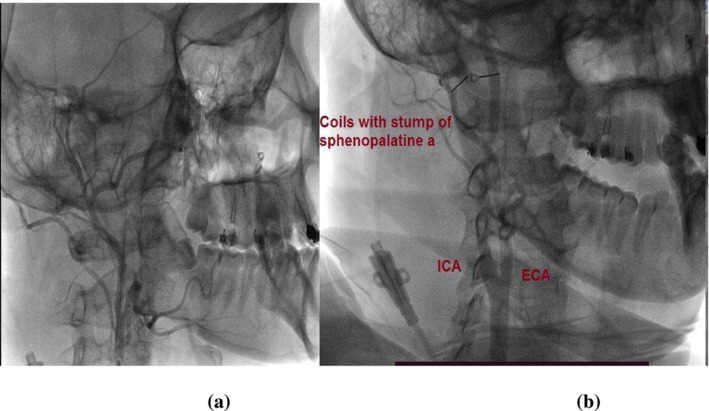
(a) selective angiography of the right maxillary artery, arterial phase, shows diffuse active bleeding in the territory of the right sphenopalatine artery. Selective embolization was performed by injecting Gelfoam fragments. (b) Angiography, arterial phase after embolization of the third portion of the right internal maxillary artery with 2 mm coils and gelfoam resulting in occlusion of the second and third portions of the right internal maxillary artery.

Approximately 1 year later, she returned with recurrent hematemesis, and an epistaxis was identified in the contralateral sphenopalatine artery. She was referred again for endovascular intervention and underwent successful selective embolization of the third portion of the right internal maxillary artery with a 2 mm coil and gel foam (Figures 2 and 3). Follow‐up revealed no complications, and her condition improved steadily. She was discharged in good general health with stable vital signs.

## 5 Discussion

Our patient was found to have posterior epistaxis after a 7‐month period of recurring hematemesis, melena, and several admissions for blood transfusions. The diagnosis of epistaxis was established after a long period of time during which she was suspected to have upper gastrointestinal bleeding (UGIB). The differential diagnosis of hematemesis and melena includes the causes of UGIB. These include erosive or inflammatory causes (like peptic ulcer disease, esophagitis, gastritis, etc.), vascular causes (varices, dieulafoy lesion, angiodysplasia, etc.), tumors (gastric cancer, esophageal cancer), traumatic or iatrogenic causes (mallory‐weiss tear, foreign body ingestion, etc.), coagulopathies, hemobilia, or hemosuccus pancreaticus [5, 6, 7].

When a patient presents with signs and symptoms of UGIB, the patient goes through many diagnostic measures. These start with laboratory studies to assess the hemoglobin status, platelet counts, liver chemistries (to assess for esophageal varices hemorrhage), and coagulation panels to look for coagulopathies [8]. Then, within 24 h, endoscopy is performed, which diagnoses many of the erosive, inflammatory causes, tumors or iatrogenic causes, hemobilia, or hemosuccus pancreaticus [9]. If the endoscopy was negative, a colonoscopy would be performed if melena or hematochezia were present because the cause might be from the lower gastrointestinal bleeding (LGIB) [10]. If all were negative, then an evaluation for small bowel bleeding should be performed [10]. There are many options for it, including CT angiography, push enteroscopy, push and pull enteroscopy, and video capsule endoscopy (VCE) [10].

When the patient is hemodynamically stable, VCE is preferred, and when the patient is hemodynamically unstable, angiography should be performed [10]. In our case, the patient underwent the process mentioned above with no clear diagnosis in any of the investigations. HEENT examination should be part of the investigations as it can help guide the diagnosis [11].

In our case, the patient's sphenopalatine bleeding was episodic. Along with the severity of the bleeding and the lack of nasal bleeding, these factors led to the delay in the diagnosis of her condition. Epistaxis can occur in people of all ages, with a bimodal distribution most commonly affecting those < 10 and older than 50 years of age. Up to 90% of epistaxis is anterior epistaxis, which refers to a nosebleed that originates from the anterior part of the nose and is common in children. The majority of anterior epistaxis cases commonly arise from the Kiesselbach plexus. The remaining 10% of epistaxis refers to bleeding from the posterior nasal cavity and is common after the age of 50. Most often, it originates from the Woodruff plexus, which is formed by anastomoses of the sphenopalatine artery and pharyngeal artery [12].

Our patient is 21 years old, and posterior epistaxis is uncommon at this age. The combination of severe bleeding and a history of peptic ulcer disease also contributed to the suspicion of a UGI source of the bleeding. The delay in the diagnosis was due to a combination of these factors. While the majority of anterior bleeding is demonstrated clinically, posterior bleeding may present with confusing symptoms such as melena, hematemesis, or anemia because blood may flow down the throat [12]. While the prognosis for epistaxis is generally good, it can be life‐threatening, as seen in our patient [13]. Overall, the percentage of patients with epistaxis requiring hospitalization ranges from 2% to 40.8% [14, 15].

It is important to evaluate the patient's airway, breathing, and circulation. When clinical instability is present, hemodynamic stabilization should be done before the cessation of epistaxis [12]. A study published in 1987 reported that 0.55% of epistaxis patients show symptoms suggesting UGIB [16]. Another study found that epistaxis was diagnosed in 4.3% of end stage liver disease (ESLD) patients admitted to the hospital for suspected serious UGIB [17].

In our case, no abnormalities or any obvious source of bleeding were diagnosed, and the patient continued to experience recurrent episodes of hematemesis; the cause was not identified by endoscopy. Treatment of posterior epistaxis typically involves a combination of many interventions. Nasal packing is often employed as an initial step to apply direct pressure and promote clotting [18]. For refractory or recurrent bleeding, arterial embolization or endoscopic ligation of the bleeding vessel is considered [18, 19]. Multidisciplinary collaboration among healthcare professionals is essential for the successful management of posterior epistaxis [19].

We found three similar cases in the literature other than the study focusing on patients with ESLD, with their data summarized in Table 1. Interestingly, two of the three patients were between the ages of 76 and 78, and one was 1 year old. These three cases are found within the bimodal age presentations of epistaxis (before 10 years and after 45 years) [20, 21].

**TABLE 1:** Represent three cases similar to our case presented with GI manifestations due to epistaxis of different etiologies.

Reference	Case	Age	Sex	Presenting symptoms	The cause of epistaxis
Yano et al. [20]	1	78	Male	Melena and shock	Nasal polyps
Wolf et al. [21]	2	76	Male	Hematemesis	Idiopathic
Wolf et al. [21]	3	1	Male	Hematemesis and melena	Idiopathic

## Author Contributions

**Aref AlRajabi:** conceptualization, data curation, formal analysis, investigation, methodology, supervision, writing – original draft, writing – review and editing. **Bara M. AbuIrayyeh:** conceptualization, data curation, investigation, writing – original draft, writing – review and editing. **Amal M. Shawabka:** conceptualization, formal analysis, methodology, writing – review and editing. **Anwar Yousef Jabari:** data curation, investigation, writing – original draft. **Sami D. Jabari:** data curation, investigation, writing – review and editing. **Kareem Ibraheem:** conceptualization, data curation, writing – original draft, writing – review and editing.

## Consent

Written informed consent was obtained from the patient for the publication of this case report.

## Conflicts of Interest

The authors declare no conflicts of interest.

## Data Availability

The data used to support the findings of this study are included in the article.
